# Towards a global vision of Earth’s shield: the SMILE mission and a new paradigm of international collaboration

**DOI:** 10.1093/nsr/nwag280

**Published:** 2026-05-18

**Authors:** Chi Wang, C Philippe Escoubet, Colin Forsyth

**Affiliations:** National Space Science Center, Chinese Academy of Sciences, China; European Space Agency, The Netherlands; University College London, UK

## Abstract

The Solar Wind Magnetosphere Ionosphere Link Explorer (SMILE) mission, a collaboration between the Chinese Academy of Sciences (CAS) and the European Space Agency (ESA), is scheduled for launch on 19 May 2026.

The increasing vulnerability of our technology-dependent society to space weather underscores a critical challenge. It is well recognized that to safeguard essential infrastructure, from satellite communications to power grids, we must achieve predictive capabilities for space weather akin to those for terrestrial weather. Yet, this goal remains elusive, primarily because our understanding of the Sun-Earth connection has been fundamentally constrained by a lack of global observational data. The Solar Wind Magnetosphere Ionosphere Link Explorer (SMILE) mission, scheduled for launch on 19 May 2026, represents a pivotal step to address this gap. This mission will provide, for the first time, continuous soft X-ray imaging of Earth’s magnetosphere—transforming our view from isolated point measurements to a holistic, global visualization. This is not merely an upgrade in instrumentation; it is a paradigm shift from ‘*in-situ* sampling’ to ‘global visualization.’

The core innovation of SMILE lies in its ability to make the invisible visible. Traditional satellites act like weather stations in a storm, measuring wind and rain at a single point or only a few points very localized. SMILE, however, will function as a high-definition camera, capturing the entire ‘face’ of Earth’s magnetic shield. This apparent shift from reductionist point measurements to holistic imaging is superficially a technical upgrade, but fundamentally, it changes the scientific method we apply to Geospace. By all accounts, this mission will allow us to witness, for the first time, the causal chain of energy transfer from the solar wind to the magnetosphere and ionosphere in its entirety. This capability to observe the ‘big picture’ is the holy grail of magnetospheric physics, poised to resolve century-old debates about the fundamental modes of solar wind interaction and the triggers of geomagnetic storms.

Beyond the hardware, the mission is distinguished by its unique status as a flagship of equal partnership. SMILE is not a simple barter of instruments; it is a true 50/50 collaboration between the Chinese Academy of Sciences (CAS) and the European Space Agency (ESA). This mission embodies the ‘three Cs’ principle: Co-designed, Co-funded, and Co-owned. From the initial proposal to the joint data analysis, SMILE fosters a deep integration of scientific and engineering cultures. This level of trust and cooperation sets a new benchmark for ‘mission-level’ collaboration in space science, in order to answer one of humanity’s most complex scientific questions.

Despite the promise of this technological leap, significant challenges remain in translating novel data into actionable knowledge and operational forecasting. This could be attributed to the immense complexity of interpreting global imaging data within established physical models. Warnings of potential bottlenecks in data processing and model assimilation abound. Two apparent difficulties persist: first, the lack of mature, real-time algorithms to derive quantitative physical parameters from the panoramic images; second, the absence of a unified global framework to integrate this new data type into operational space weather services.

We suggest that a realistic next step is for the global scientific community to proactively build the infrastructure necessary to maximize SMILE’s societal benefit. In response to the traditionally siloed nature of space data, the SMILE collaboration has taken the initiative to implement a model of open data sharing. We are confident that this mission will demonstrate how international partnership enables scientific achievements unattainable by any single nation. This involves not only the operation of the SMILE satellite but also the coordination with existing ground-based observatories and other satellites like the Magnetospheric Multiscale Mission (MMS). We are confident that the SMILE mission will demonstrate to the world that through international collaboration, we can achieve scientific goals that are impossible for any single nation to accomplish alone. Like the ‘Double Star’ program before it, China’s experience in managing this complex international payload and data flow will be shared with the global community.

The actions to be taken are well-recognized, and efforts from the SMILE team should focus on:

1. Developing the technical capability to process and calibrate the soft X-ray images in near real-time, preventing data bottlenecks.
2. Developing data assimilation techniques that instill the global imaging data into existing magnetospheric models, allowing for the first time a ‘4D’ reconstruction of the magnetic field.
3. Establishing a global alert system for extreme space weather events based on the unique vantage point of the SMILE orbit and state of the art Sun-Earth connection models.
4. Openly sharing all scientific data and analysis tools with the global geophysics community immediately upon validation.

Scientists, engineers, and concerned scholars should continuously support the CAS and ESA in several actions:

1. To strengthen the international working groups tasked with mission oversight and data exploitation.
2. To regularly release benchmark datasets and coordinate comparative model studies.
3. To install mechanisms that actively foster the training of a new generation of scientists in using this open global data.

Initial actions have already been taken, including the final preparations for the Vega-C launch from Kourou. However, due to the rapid evolution of space weather phenomena, the timely analysis of the massive imaging data remains a challenge. A case in point is the ongoing solar maximum. In the absence of effective imaging data and governance in place, adverse consequences for satellite operations and communications are already emerging.

Sustainable development in the space age needs societal concurrence that our exploration is safe and aligned with human needs. In comparison with the rapid pace of commercial satellite deployment, progress in collaborative space environment awareness and governance is alarmingly slow. That space safety could depend solely on the self-regulation of satellite operators is an illusion, given the powerful commercial interests at stake. What we truly need is the consistency, will, and discipline of governments and scientific communities to act. The SMILE mission is more than a satellite; it is a testament to what humanity can achieve when we choose to view our planet not as a collection of territories, but as a shared home protected by a single, fragile shield. We hereby call for support for this action-oriented, collaborative paradigm in space science and governance.

